# Long noncoding RNA BLACAT1 indicates a poor prognosis of colorectal cancer and affects cell proliferation by epigenetically silencing of p15

**DOI:** 10.1038/cddis.2017.83

**Published:** 2017-03-09

**Authors:** Jun Su, Erbao Zhang, Liang Han, Dandan Yin, Zhili Liu, Xuezhi He, Yuhong Zhang, Feng Lin, Qingfeng Lin, Peiyao Mao, Weidong Mao, Dong Shen

**Affiliations:** 1 Department of Oncology, The Affiliated Jiangyin Hospital of Southeast University Medical College, Jiangyin, Jiangsu, China; 2 Department of Epidemiology and Biostatistics, Collaborative Innovation Center For Cancer Personalized Medicine, School of Public Health,Nanjing Medical University, Nanjing, China; 3 Jiangsu Key Lab of Cancer Biomarkers, Prevention and Treatment, Collaborative Innovation Center of Cancer Medicine, Nanjing Medical University, Nanjing, China; 4 Department of Oncology, Xuzhou Central Hospital, Affiliated Xuzhou Hospital, College of Medicine, Southeast University, Xuzhou, Jiangsu, China; 5 Central Laboratory, The Second Affiliated Hospital of Southeast University, Nanjing, Jiangsu, China; 6 Department of Biochemistry and Molecular Biology, Nanjing Medical University, Nanjing, Jiangsu, China; 7 Department of Clinical Medicine, Nanjing Medical University, Nanjing, Jiangsu, China

## Abstract

Recently, a novel class of transcripts, long noncoding RNAs (lncRNAs), is being identified at a rapid pace. These RNAs have critical roles in diverse biological processes, including tumorigenesis. One of them, BLACAT1, a cancer-associated long noncoding RNA, exerts regulatory functions in various biological processes in cancer cells, however, the role of BLACAT1 in colon cancer remains unclear. Our experiments showed that increased BLACAT1 was an independent unfavorable prognostic indicator for colorectal cancer, and revealed that BLACAT1 knockdown significantly repressed proliferation, both *in vitro* and *in vivo*. Mechanistic investigations demonstrated that BLACAT1 had a key role in G1/G0 arrest, and showed that BLACAT1 can repress p15 expression by binding to EZH2, thus contributing to the regulation of CRC cell cycle and proliferation. Our results suggest that BLACAT1, as a cell cycle regulator, may serve as a potential target for colon cancer prevention and treatment in human CRC.

Colorectal cancer is a common cause of cancer death in the world due to late tumor presentation and rapid progression, with about 13 million new cancer cases and 694000 cancer deaths in 2012 worldwide.^1, 2^ China is one of the countries with high incidence of CRC, in which CRC ranks third among all cancer sites in cancer incidence and fifth in cancer mortality.^3^ CRC incidence and mortality rates are continuing to rise in China, the 5-year survival rate for colorectal cancer is estimated to be 30% in India, China and Cuba.^4^ CRC is rapidly becoming a main public health problem. With the deepening of research, colorectal cancer is now considered a heterogeneous group of diseases with distinct clinical, pathological and molecular features, which results from the dysregulation of many tumor-related genes. Therefore, a better understanding of the molecular machanisms on CRC carcinogenesis and progression may supply more options for the treatment and to improve the prognosis of CRC patients. Currently, accumulating evidence has shown that noncoding RNAs (ncRNAs) may be involved in CRC pathogenesis,^5, 6, 7, 8, 9^ providing new insights into the biology of this cancer.

It is well known that less than 2% of the mammalian genome are protein-coding genes and over 90% of genome represent noncoding RNAs (ncRNA), which are transcribed but not encode proteins. Despite the initial controversy regarding their biological characters, increasing evidence had showed that ncRNA are highly regulated and functional.^10, 11, 12, 13^ Long intergenic ncRNAs (lincRNA) range in size from several hundred to tens of thousands of bases (⩾200). They belong to a newly discovered class of ncRNAs. Although more than 3000 human lincRNAs have been identified, less than 1% of them have been characterized.^14^ Long noncoding RNAs were initially thought to be spurious transcriptional noise but are emerging as new regulators in the cancer paradigm.^15^ In fact, emerging evidence indicates that IncRNAs may have complex and extensive functions in the development and progression of cancer. For example, HOTAIR, functioning as an oncogene, that can be used to predict the prognosis in non-small cell lung cancer (NSCLC) patients and determine whether or not patient can benefit from chemotherapy.^16, 17^ GAS1 that downregulated in papillary thyroid cancer leaded to the activation of PI3K/Akt/Bad pathway and the promotion of proliferation.^18^ In colorectal cancer, MALAT1 and 91H can be used as predictive biomarker for poor prognosis,^19, 20^ UCA1 influences cell proliferation and apoptosis of colorectal cancer.^21^

BLACAT1 (linc-UBC1) is one of the few well-known lincRNAs, with a length of 2616 bp and a functional role in recruiting and binding to polycomb repressive complex 2 (PRC2), first characterized in bladder cancers.^22^ Recent studies suggest BLACAT1 exhibited tumor pro-oncogenic activity in gastric cancer, and additionally may serve as an negative predictor for prognosis in GC patients.^23^ The biological roles of BLACAT1 in colorectal cancer have not been well understood, which prompted us to explore the functions of BLACAT1 in human colorectal cancer.

In this study, we detected the expression pattern of BLACAT1 in CRC tissues and matched adjacent nontumor tissues, and analyzed the correlation with clinicopathological factors of patients. We also explored the function of BLACAT1 by using *in vitro* and *in vivo* assays, and further researched the interaction between PRC2 and BLACAT1 to explore the epigenetic repression of cyclin-dependent protein kinase inhibitors(CKIs), including P15, which may partly account for BLACAT1-mediated proliferation regulation, thus affecting the proliferation of CRC.

## Results

### BLACAT1 expressions are upregulated in CRC tumor tissues and significantly correlated with TNM stage

**Figure 1:**
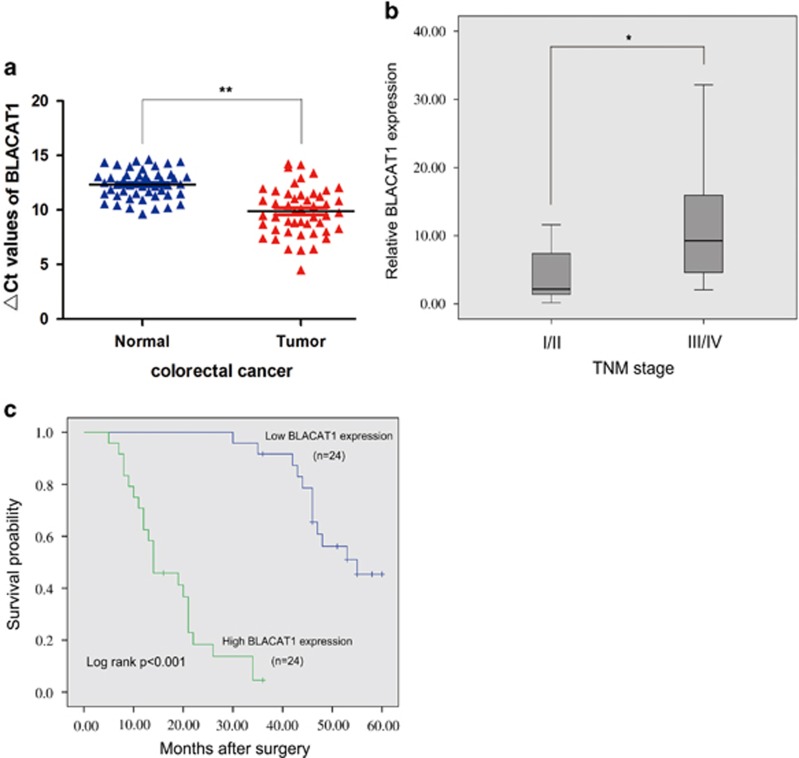
Expression of BLACAT1 in CRC tissues and its clinical parameters. (**a**) Relative expression of BLACAT1 in CRC tissues (*N*=48) campared with the corresponding non-tissues (*N*=48). BLACAT1 expression was examined using quantitative real-time PCR(qRT-PCR) and normalized to GAPDH expression. The ΔCt value was determined by subtracting the GAPDH Ct value from the BLACAT1 Ct value. Smaller ΔCt value indicates higher expression. (**b**) Higher BLACAT1 was positively correlated with TNM stage. (**c**) Patients with high levels of BLACAT1 expression showed reduced survival times compared with patients with low levels of BLACAT1. *P<0.05, **P<0.01

First, we examined the BLACAT1 level in the CRC tumor tissues. The expression of BLACAT1 in tumor tissues relative to adjacent normal tissues is shown in Figure 1. Among all the 48 pairs of CRC patients, BLACAT1 expression levels in tumors were higher than those in the corresponding normal tissues on the whole (12.2732±24.99594, *P*<0.001; Figure 1a). Next, we explored the correlation of BLACAT1 expression level with the clinicopathological factors in CRC patients. The results demonstrated that the BLACAT1 level was remarkably correlated with TNM stage. Advanced TNM stage was significantly positively related to increased BLACAT1 expression, showing 18.6586±32.64750 *versus* 4.7268±4.39707 (*P*=0.041; Figure 1b). In this study, BLACAT1 expression of tumors infiltration confined within the mucosal layer (so-called early colorectal cancer) was compared with that of tumor infiltration beyond the mucosal layer. Univariate and multivariate analysis show that BLACAT1 expression is an independent predictor for overall survival.

To further evaluate the value of BLACAT1 in prognosis of patients with CRC, we used Kaplan–Meier survival analysis and log-rank tests. We divided the samples into high (above the mean, *n*=24) and low (below the mean, *n*=24) BLACAT1 expression groups according to the mean value of the BLACAT1 level. As shown in Figure 1c, increased BLACAT1 level was associated with shorter overall survival (*P*<0.001).

Univariate analysis identified three prognostic factors: distant metastasis (M0, M1), TNM stage (I/II, III/IV) and BLACAT1 expression. The other clinicopathological characteristics, such as tumor invasion depth, histological grade, lymph node metastasis, patients' age and sex were not statistically significant prognosis factors. Multivariate analysis further revealed that BLACAT1 expression was a significant independent predictor of poor survival of CRC patients (*P*<0.001; Table 1).

### Manipulation of BLACAT1 levels in CRC cells

**Figure 2:**
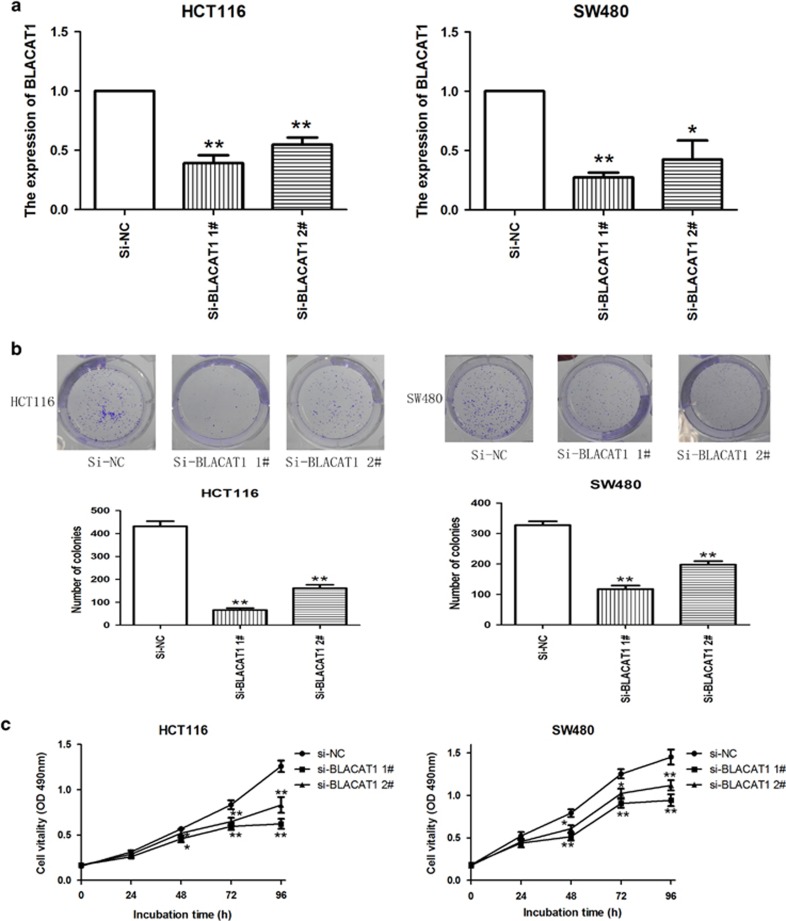
BLACAT1 regulates CRC cell proliferation *in vitro*. (**a**) The results of qPCR determined that the relative expression level of BLACAT1 decreased by transfection with si-BLACAT1 in CRC cells, compared with the transfection with si-NC. (**b**) A colony-formation assay revealed that BLACAT1 knockdown suppressed colony formation, compared with control cells. (**c**) MTT assays indicated that decreased BLACAT1 expression inhibited HCT116 and SW480 cells proliferation. *P<0.05, **P<0.01

In our research, we chose SW480 and HCT116 cells. To manipulate the BLACAT1 level in CRC cells, BLACAT1 siRNA was transfected into HCT116 and SW480 cells, two effective interference target sequences of BLACAT1 were used. After transfection, we examined the BLACAT1 levels by qRT-PCR. The expression of BLACAT1 significantly reduced in si-BLACAT1-transfected CRC cells (Figure 2a).

### Effect of BLACAT1 on CRC cell proliferation, apoptosis and cell cycle *in vitro*

**Figure 3:**
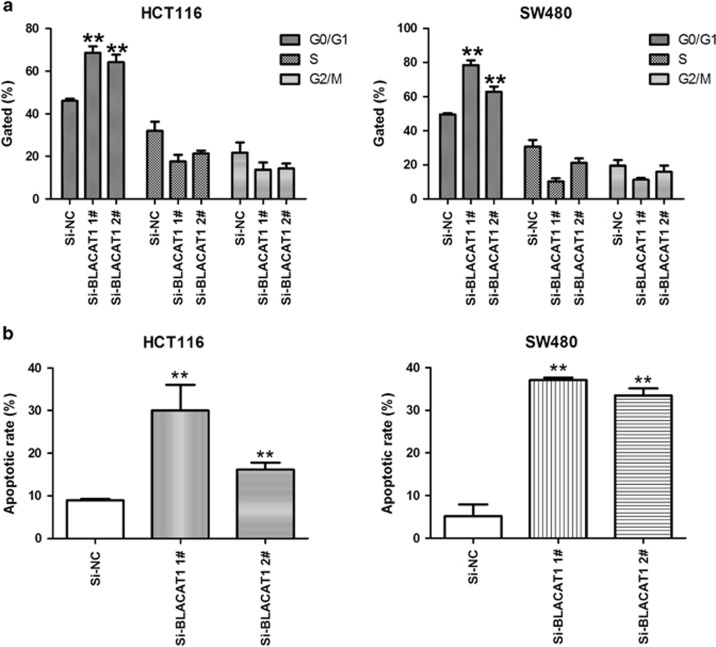
BLACAT1 is a regulator of gastric cancer cell cycle. (**a**) Flow cytometry showed that cell cycle arrest at the G1–G0 phase increased in both HCT116 and SW480 cells when BLACAT1 was repressed. (**b**) The results of apoptosis assays indicated that knockdown of BLACAT1 could obviously induce cell apoptosis. Error bars indicate means±S.E.M. **P<0.01

To assess the function of BLACAT1 in CRC, we investigated the effect of targeted knockdown of BLACAT1 on cell proliferation and apoptosis. Colony-formation assays indicated that clonogenic survival was significantly decreased in si-BLACAT1-transfected HCT116 and SW480 cells (Figure 2b), Similarly, the results of MTT assays revealed that cell growth was inhibited following downregulation of BLACAT1 in si-BLACAT1-transfected CRC cells compared with respective controls (Figure 2c). Next, flow cytometric analysis was performed to further examine the effect of BLACAT1 on CRC cells. si-BLACAT1 HCT116 and SW480 cells were significantly stalled at G1–G0 phase, compared with si-NC (Figure 3a). And our results demonstrated a significantly higher percentage of apoptosis cells for si-BLACAT1 transfected HCT116 and SW480 cells, while compared with si-NC-treated cells (Figure 3b). Taken together, these results indicate that downregulation of BLACAT1 inhibit CRC cells proliferation, and induce cell apoptosis *in vitro*.

### The impact of BLACAT1 *in vivo*

**Figure 4:**
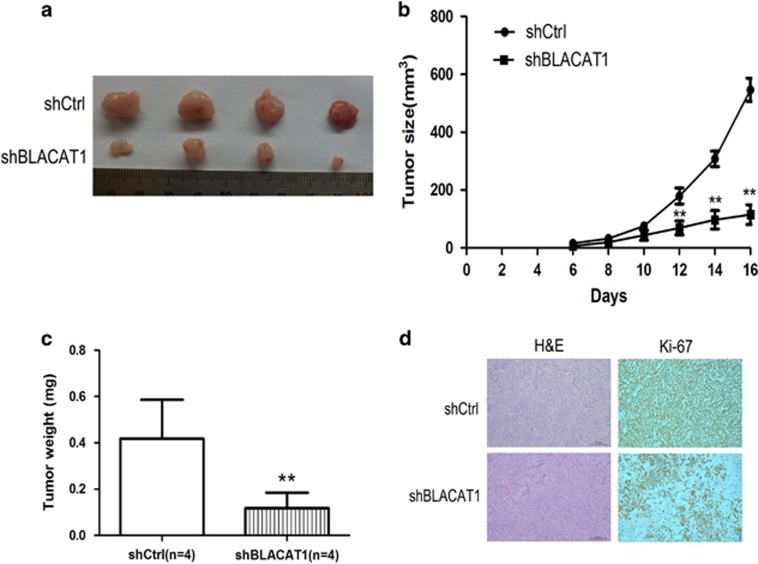
The impact of BLACAT1 on tumorigenesis *in vivo*. (**a** and **b**) Scramble or shBLACAT1 was transfected into HCT116 cells, which were injected into nude mice (*N*=4). The tumor volumes were calculated every 2 days after injection. The bars indicate S.D. (**c**) The tumor weights were lower than that in the control group. (**d**) Histopathology of xenograft tumors. The tumor sections underwent H&E staining and IHC staining using antibodies against Ki-67. Bar, 100 *μ*m. Error bars indicate means±S.E.M. **P<0.01

To further determine whether the BLACAT1 affects tumorigenesis *in vivo*, we injected shCtrl or shBLACAT1-transfected HCT116 cell into nude mice. Consistent with *in vitro* results, the growth of CRC cells in shBLACAT1 group was significantly slower than in the control group (Figures 4a and b). Up to 16 days after injection, the average tumor weight in the shBLACAT1 group was obviously lower than that in the control group (Figure 4c). IHC analysis was performed to detect the expression of Ki-67 in tumor tissues, we found that the tumors formed from the shCtrl group showed stronger Ki-67 expression than that formed from shBLACAT1 (Figure 4d).

### BLACAT1 was required for the epigenetic repression of p15 by binding to PRC2, thus contributing to the regulation of the CRC cell cycle and cell proliferation

**Figure 5:**
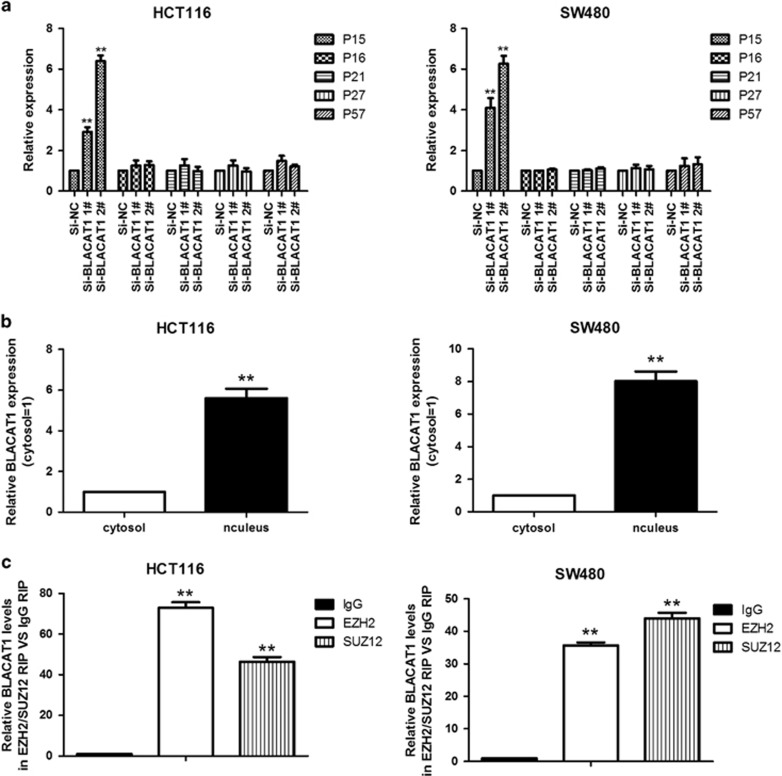
BLACAT1 is associated with PRC2 in CRC. (**a**) The expression of p15, p16, p21, p27 and p57 were determined after knockdown of BLACAT1 using qRT-PCR. (**b**) BLACAT1 nuclear localization, as identified using qRT-PCR in fractionated HCT116 and SW480 cells. After nuclear and cytosolic separation, RNA expression levels were measured by qRT-PCR. GAPDH was used as a cytosolic marker. (**c**) RIP experiments were performed, and the co-precipitated RNA was subjected to qRT-PCR for BLACAT1. The fold enrichment of BLACAT1 in RIPs is relative to its matching lgG control RIP. **P<0.01

To probe the fact that BLACAT1 have a role in GO/G1 arrest, we researched the expression of inactivation of CDK inhibitors (CKIs), and the results indicated that p15 was significantly increased with knockdown of BLACAT1, compared with control group (Figure 5a). To further study the mechanism of BLACAT1 in the role of CRC cell cycle, we detected the expression of BLACAT1 in nucleus *versus* cytosol by qRT-PCR, the results demonstrated that BLACAT1 expression was obviously higher in nucleus than cytosol (Figure 5b), suggested that BLACAT1 may have a major regulatory function at the transcriptional level. He *et al.*^22^ found that BLACAT1 (linc-UBC1) could physically associate with PRC2 complex and regulates histone modification status of target genes in bladder cancer. We researched the correlation between PRC2 and BLACAT1 by RNA immunoprecipitation (RIP) in colorectal cancer cells, then we observed that endogenous BLACAT1 was significantly enriched in the anti-EZH2 RNA-IP fraction. Similarly, the endogenous BLACAT1 was enriched in the anti-SUZ12 RIP fraction relative to the input compared with the IgG fraction both in HCT116 and SW480 cell lines (Figure 5c).

**Figure 6:**
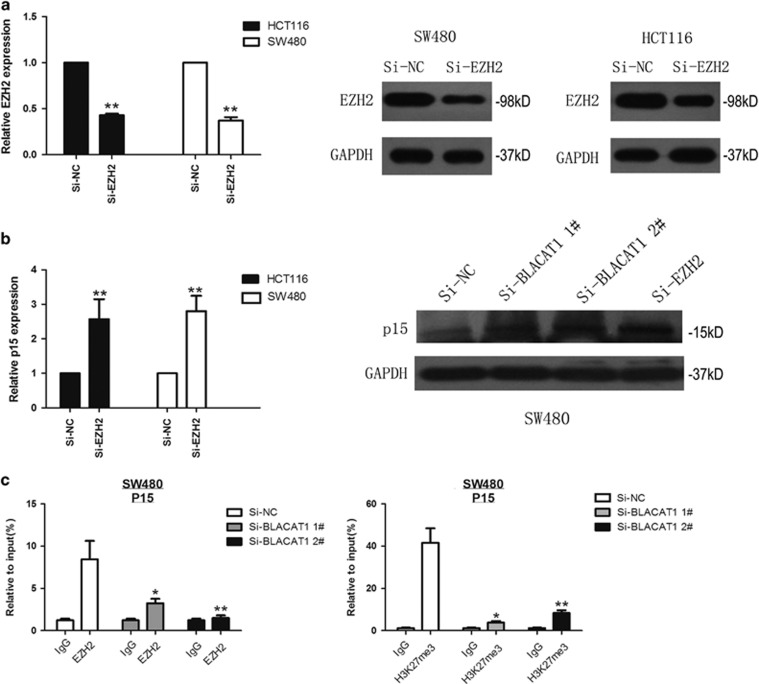
BLACAT1 is required to target PRC2 occupancy and activity to epigenetically regulate the expression of CKIs, thus regulating gastric cancer cell cycle and proliferation. (**a**) The expression of EZH2 was determined after knockdown of EZH2 by qRT-PCR and western blot assays. (**b**) The expression of p15 was determined after knockdown of EZH2 by qRT-PCR and western blot assays, and relative expression was determined in SW480 cells transfected with si-BLACAT1 (si-BLACAT1 1# and si-BLACAT1 2#) by western blot assays. (**c**) ChIP-qPCR of H3K27me3 and EZH2 of the promoter region of p15 locus after siRNA treatment targeting si-NC or si-BLACAT1 in SW480 cells, Antibody enrichment was quantified relative to the amount of input DNA. Antibody directed against IgG was used as a negative control. **P<0.01

Next, to address the role of PRC2 in co-regulating the suppression of BLACAT1-suppressed p15, we inhibited the expression of EZH2 in HCT116 and SW480 cells by tranfection with siEZH2 (Figure 6a), and detected the p15 expression by qRT-PCR and western blot. As shown in Figure 6b, the expression of p15 was upregulated after knockdown of BLACAT1 and EZH2.

To illustrate whether BLACAT1 is involved in transcriptional supression through the enrichment of EZH2 to target gene promoter of p15, we performed chromatin immunoprecipitation (ChIP) analysis after BLACAT1 knockdown. The ChIP assays indicated that knockdown of BLACAT1 decreased the binding of EZH2 and H3K27me3 levels across the promoter of p15 (Figure 6c). The levels of EZH2 were not affected by BLACAT1-knockdown cells. These results demonstrated that the reduction in PRC2 chromatin binding and H3K27me3 are mediated by BLACAT1 knockdown. These results suggest that BLACAT1 is required to target EZH2 occupancy and works to epigenetically regulate the expression of p15.

## Discussion

CRC is a group of diverse heterogeneous diseases arising through various molecular pathways. This heterogeneity determines tumor prognosis and response to therapy and brings great challenges not only in studying the molecular basis of the disease, but also in clinical patient management. To date, there are few reliable markers available to accurately predict prognosis of CRC, some molecular markers are in use for treatment decisions and patient stratification, such as the mutation status of BRAF V600E, NRAS and KRAS,^24, 25, 26^ that still have many insufficiency. So there is a strong need to identify novel biomarkers that better conduct the option of treatments and predict overall survival of CRC patients.

Currently, lncRNAs as a novel molecular star have been increasingly reported to be involved in human disease, especially in cancers.^12, 27, 28, 29^ Previous studies indicated that BLACAT1 is overexpressed in bladder cancer, gastric cancer^22, 23^ and acted as the negative prognostic factor. However, the expression and roles of BLACAT1 in CRC are not being elucidated. In our present study, we found that the average level of BLACAT1 in CRC tissues was significantly higher than in corresponding nontumor tissues. The high expression level of BLACAT1 in CRC patients was positively correlated with TNM stage. Moreover, high BLACAT1 expression in CRC tissues was associated with a poor prognosis and could be an independent prognostic indicator. These results suggested that BLACAT1 might have an important role in CRC progression.

To further analyze the role of BLACAT1 in CRC, RNA interference approaches were used. As a result, inhibition of BLACAT1 could promote CRC cell proliferation both *in vitro* and *in vivo*. And, the knockdown of BLACAT1 could induce obvious G0/G1 arrest and cause apoptosis. Previous studies have reported that many lncRNAs recruit PRC2 complexes to target genes, and PRC2-mediated epigenetic regulation has an important role in cancer. Twenty percent of all human lncRNAs have been shown to physically associate with Polycomb Repressive Complex 2 (PRC2 complex), suggesting that lncRNAs may have a universal role in recruiting polycomb-group proteins to their target genes.^30^ The previous research confirmed that BLACAT1(linc-UBC1) physically associates with PRC2 complex and regulates histone modification status of target genes in bladder cancer.^22^ Our results suggest that BLACAT1 serves as a member of PRC2-mediated epigenetic regulation and may participate in the occurrence and development of CRC.

As we know, the kinase activity of Cdk/cyclin complexes is closely modulated by a plethora of Cdk inhibitors (CKIs), which serve as brakes to halt cell cycle progression.^31^ In addition, CKIs act as tumor suppressors in various cancers, and aberrant methylation in the CKI gene promoter region has been linked to downregulation of gene expression,^32^ while PRC2-mediated histone methylation contributes to the repression of CKIs.^33, 34, 35, 36, 37^ Thus, we researched the effect of the knockdown of BLACAT1 and EZH2 on the expression of several CKI family proteins involved in the G1/S checkpoint in HCT116 and SW480. Our results illustrated that the knockdown of BLACAT1 could obviously induce the expression of P15 (one of CKIs family members) in an EZH2-dependent manner.

Our study identified EZH2 as having an important role in this BLACAT1-mediated p15 repression network because the ability of BLACAT1 to repress p15 was dependent on the association between BLACAT1 and EZH2. As shown in Figure 5b, analysis with qRT-PCR and western blotting confirmed that the expression of p15 was increased when EZH2 was knocked down. In our research, we illustrated high abundance binding between BLACAT1 and EZH2 in colorectal cancer cells, and we further confirmed that BLACAT1 could mediate epigenetic regulation of p15. As shown in Figure 5c, ChIP-qPCR assays determined that BLACAT1 was required for the EZH2 recruitment and silencing of p15. Our study explained how CKIs are specifically regulated by PRC2, in part due to BLACAT1. These results demonstrated that BLACAT1 could have a key role in the cell cycle of colorectal cancer.

In a broader perspective, the identification of BLACAT1 as an important prognostic factor for CRC patients attract our attention to exploring its functional roles. Moreover, BLACAT1 could regulate colorectal cancer cells proliferation both *in vitro* and *in vivo*. Importantly, we first reported that BLACAT1 serving as a member of PRC2-mediated epigenetic regulation participated in the development of colorectal cancer. Our research may provide a strategy and facilitate the development of lncRNA-directed diagnostics and therapeutics against this deadly disease.

## Materials and methods

### Clinical samples

A total of 48 fresh cancer tissue samples and matched adjacent nontumor tissues were obtained from stage I/II and III/IV CRC (according to the seventh version of the American Joint Committee on Cancer staging system) patients who underwent surgical resection without preoperative chemotherapy or radiotherapy at the Affiliated Jiangyin Hospital of Medical College of Southeast University between May 2010 and November 2011. All the specimens were immediately frozen in tubes containing RNAlater preservation liquid after removal and stored at liquid nitrogen until RNA extraction. The tumor samples were pathologically confirmed by pathologists.The clinicopathological characteristics of the CRC patients are summarized in Table 2.

### Cell lines and culture conditions

Two CRC adenocarcinoma cell lines (HCT116, SW480) were purchased from the Institute of Biochemistry and Cell Biology of the Chinese Academy of sciences (Shanghai, China). All the cell lines were cultured in DMEM (GIBCO-BRL) medium supplemented with 10% fetal bovine serum (FBS) in humidified air with 5% CO2 at 37 °C.

### RNA extraction and quantitative real-time PCR

Total RNA was extracted from tissues or cultured cells with TRIzol reagent (Invitrogen, Grand Island, NY, USA) according to the manufacturer's protocol. qRT-PCR assays were performed to detect BLACAT1 expression using the PrimeScript RT reagent Kit and SYBR Premix Ex Taq (Takara, Dalian, China) according to the manufacturer's instructions. The results were normalized to the expression of glyceraldehydes-3-phosphate dehydrogenase (GAPDH). The primers used were as follows: BLACAT1 sense, 5′-GTCTCTGCCCTTTTGAGCCT-3′ and antisense, 5′-GTGGCTGCAGTGTCATACCT-3′. qRT-PCR and data collection were performed on an ABI 7500. The relative expression of BLACAT1 was calculated and normalized using the 2^−ΔΔCt^ method relative to GAPDH.

### Cell transfection

Three individual small interfering RNA (siRNA) and scrambled negative control siRNA (si-NC) were purchased from Invitrogen. The target sequences for BLACAT1 siRNAs were as follows: (si-BLACAT1① 5′-AGGCUGGUUUCUGCCCUCAUCCUUU-3′, si-BLACAT1②, 5′-GCCCAGCUUCUAGUCCUCUCCUUAU-3′) BLACAT1 siRNA or si-NC were transfected into HCT116 and SW480 cells. SW480 and HCT116 cells were grown on six-well plates to confluency and transfected using Lipofectamine 2000 (Invitrogen) according to the manufacturer's instructions. Forty-eight hours after transfection, the cells were collected for qRT-PCR or western blot analyses.

### Cell proliferation assays

The cell proliferation was analyzed using Cell Proliferation Reagent Kit I (MTT; Roche Applied Science, Basel, Switzerland). Si-BLACAT1-transfected SW480 and HCT116 cells (2000/well) were allowed to grow in 96-well plates. Cell proliferation was documented every 24 h following the manufacturer's protocol. All the experiments were performed in quadruplicate. For the colony-formation assay, a total of 500 BLACAT1 siRNA-ttansfected HCT116 and SW480 cells were placed in a fresh six-well plate and maintained in media containing 10% FBS, replacing the medium every 3 days. After 14 days, the cells were fixed with methanol and stained with 0.1% crystal violet (Sigma-Aldrich, St. Louis, MO, USA). Visible colonies were manually counted. For each treatment group, wells were assessed in triplicate.

### Flow cytometric analysis of apoptosis

The HCT116 and SW480 cells, transiently transfected with si-BLACAT1 or si-NC, were collected 48 h after transfection by trypsinization. After double staining with FITC-Annexin V and propidium iodide, the cells were analyzed by flow cytometry (FACScan; BD Biosicences, Franklin Lakes, NJ, USA). The cells were discriminated into viable cells, dead cells, early apoptosis cells and apoptosis cells, and then the relative ratio of early apoptotic cells was compared with the control from each experiment. All the samples were assayed in triplicate.

### Western blot analysis and antibodies

The cells were lysed using RIPA protein extraction reagent (Beyotime, Beijing, China) supplemented with a protease inhibitor cocktail and phenylmethylsulfonyl fluoride (Roche, Basel, Switzerland). Protein concentration was measured using the Bio-Rad protein assay kit. Approximately 50 μg of protein extract was separated by 10% SDS-polyacrylamide gel electrophoresis (SDS-PAGE), then transferred to nitrocellulose membrane (Sigma) and incubated with specific antibodies. ECL chromogenic substrate was used to visualize the bands and the intensity of the bands was quantified by densitometry (Quantity one software; Bio-Rad, Hercules, CA, USA). GAPDH was used as a control. Antibodies (1:1000) for p15, EZH2 were purchased from Cell Signaling Technology (Boston, MA, USA).

### Tumor-formation assay in a nude mouse model

Five-week-old athymic BALB/c mice were maintained under specific pathogen-free conditions and manipulated according to protocols approved by the Shanghai Medical Experimental Animal Care Commission. The HCT116 cells were transfected with Scramble or shBLACAT1. After 48 h, the cells were collected and injected into either side of the posterior flank of the nude mouse. The tumor volumes were examined every 2 days when the implantations started to grow. The tumor volumes (length × width2 × 0.5) and weights were measured every 2 days in mice from the control (four mice) or shBLACAT1 (four mice) groups. Sixteen days after injection, the mice were killed and the tumor weights were measured.

### Subcellular fractionation location

The separation of the nuclear and cytosolic fractions was performed using the PARIS Kit (Life Technologies, Carlsbad, CA, USA) according to the manufacturer's instructions.

### ChIP assays

The ChIP assays were performed using the EZ-CHIP KIT according to the manufacturer's instructions (Millipore, Billerica, MA, USA). EZH2 and SUZ12 antibodies were obtained from Abcam (Hercules, CA, USA). H3 trimethyl Lys 27 antibody was purchased from Millipore. Quantification of immunoprecipitated DNA was performed using qPCR with SYBR Green Mix (Takara). The ChIP data were calculated as a percentage relative to the input DNA using the equation 2[Input Ct−Target Ct] × 0.1 × 100.

### RNA immunoprecipitation

RIP experiments were performed using a Magna RIP RNA-Binding Protein Immunoprecipitation Kit (Millipore) according to the manufacturer's instructions. Antibodies for RIP assays against EZH2 and SUZ12 were purchased from Abcam.

### Statistical analysis

Statistical analysis was performed using the SPSS software package (version 17.0, SPSS Inc, Armonk, NY, USA) and GraphPad Prism 5 (GraphPad Software, La Jolla, CA, USA). Statistical significance was tested by a Student's *t*-test or a Chi-square test as appropriate. Survival analysis was performed using the Kaplan–Meier method, and the log-rank test was used to compare the differences between patient groups. Statistics with *P*-value <0.05 were considered as statistically significant.

## Figures and Tables

**Table 1:** Univariate and multivariate analysis of clinicopathological factors for overall survival in 48 patients with CRC

**Risk factors**	**Univariate analysis**			**Multivariate analysis**		
	**HR**	***P*-value**	**95% CI**	**HR**	***P*-value**	**95% CI**
BLACAT1 expression	1.407	<0.001**	1.259~1.572	1.496	<0.001**	1.316~1.701
Distant metastasis (M0, M1)	25.773	<0.001**	5.056~131.361	0.12	0.172	0.006~2.517
TNM stage (I/II, III/IV)	2.022	0.046*	1.011~4.045	0.583	0.193	0.259~1.312
Lymph node metastasis (N0, N1 or above)	1.813	0.089	0.913~3.601			
Histological grade (low, middle or high)	1.963	0.207	0.689~5.587			
Age (⩽60, >60)	0.537	0.081	0.268~1.079			
Tumor invasion depth (T1, T2 or above)	2.041	0.181	0.718~5.804			
Sex (male, female)	0.947	0.876	0.479~1.873			

Abbreviation: HR, hazard ratio

*P<0.05, **P<0.01

**Table 2:** The clinicopathological factors of 48 CRC patients

**Clinical factors**	**Number of cases (%)**	**(%) of patients**
*Sex*		
Male	27	56.3
Female	21	43.8
		
*Age*		
⩽60	27	56.3
>60	21	43.8
		
*Histological grade*		
High	2	4.2
Middle	5	10.4
Middle to low	9	18.8
low	32	66.7
		
*Tumor invasion depth*		
T1	8	16.7
T2	9	18.8
T3	18	37.5
T4	13	27.1
		
*Lymph node metastasis*		
N0	23	47.9
N1	13	27.1
N2	10	20.8
N3	2	4.2
		
*TNM stage*		
I	5	10.4
II	17	35.4
III	23	47.9
IV	3	6.3
